# Influencing factors of interprofessional collaboration in multifactorial fall prevention interventions: a qualitative systematic review

**DOI:** 10.1186/s12875-023-02066-w

**Published:** 2023-05-16

**Authors:** J. S. C. Muusse, R. Zuidema, M. C. van Scherpenseel, S. J. te Velde

**Affiliations:** 1 Physical Therapy Sciences, Program in Clinical Health Sciences, University Medical Center Utrecht, Utrecht University, Utrecht, The Netherlands; 2 Research Centre for Healthy and Sustainable Living, Research Group Proactive Care for Elderly People Living at Home, HU University of Applied Sciences Utrecht, Utrecht, The Netherlands; 3 Research Centre for Healthy and Sustainable Living, Research Group Innovation of Human Movement Care, HU University of Applied Sciences Utrecht, Utrecht, The Netherlands

**Keywords:** Multifactorial fall prevention interventions, Interprofessional collaboration, Influencing factors, Community-dwelling older adults, Systematic qualitative review

## Abstract

**Background:**

With the ageing population worldwide, falls are becoming a severe and growing health problem. Interprofessional multifactorial fall prevention interventions (FPIs) have effectively prevented falls in community-dwelling older adults. However, the implementation of FPIs often fails due to a lack of interprofessional collaboration. Therefore, gaining insight into the influencing factors of interprofessional collaboration in multifactorial FPI’s for older adults living in the community is essential. Consequently, our aim was to provide an overview of factors influencing interprofessional collaboration in multifactorial FPIs for community-dwelling older adults.

**Methods:**

This qualitative systematic literature research was performed according to the Preferred Reporting Items for Systematic Reviews and Meta-Analyses (PRISMA) statement. Pubmed, CINAHL, and Embase electronic databases have been systematically searched for eligible articles, with a qualitative design. The quality was appraised using the Checklist for Qualitative Research by the Joann Briggs Institute. The findings were inductively synthesized using a meta-aggregative approach. Confidence in the synthesized findings was established using the ConQual methodology.

**Results:**

Five articles were included. Analysis of the included studies resulted in 31 influencing factors for interprofessional collaboration, which were labelled as findings. These findings were summarized in ten categories and combined into five synthesized findings. Results showed that communication, role clarity, information sharing, organization, and interprofessional aim influence interprofessional collaboration in multifactorial FPIs.

**Conclusions:**

This review provides a comprehensive summary of findings on interprofessional collaboration, specifically in the context of multifactorial FPIs. Knowledge in this area is considerably relevant given the multifactorial nature of falls, which demands an integrated, multidomain approach, including both health and social care. The results can be utilized as a fundament for developing effective implementation strategies aiming to improve interprofessional collaboration between health and social care professionals working in multifactorial FPIs in the community.

**Supplementary Information:**

The online version contains supplementary material available at 10.1186/s12875-023-02066-w.

## Background

According to the World Health Organization, falls are the second leading cause of unintentional injury deaths worldwide, with persons over 60 years old having the highest number of falls [1]. In 29 per cent of community-dwelling older adults, falls occur at least once a year, with rising numbers with increasing age [2, 3]. Falls frequently result in hip or other severe fractures [4]. Moreover, falls often have a negative impact on activities in daily living, independence, fear, and overall health outcomes [5–7]. Given the increasing older population worldwide and the growing prevalence of multimorbidity, and frailty [8], fall rates and medical expenditures are likely to increase [9, 10].

The causus of falls among older adults are multifactorial, and several risk factors have been identified [11]. These risk factors contain a combination of intrinsic factors (e.g. impaired balance, strength and gait) and extrinsic risk factors (e.g. home hazards and poor footwear) [12–15]. Given the multifactorial nature of falls, multifactorial fall prevention interventions (FPIs) appear to be the most appropriate in reducing falls [16–20]. Multifactorial FPIs target present and modifiable risk factors for falling and consist of two or more intervention components across two or more domains, such as environmental modification, medication review, and they should at least include physical exercise therapy [21, 22]. Since a single discipline will never be able to identify and manage all multifactorial risk factors for falls fully, interprofessional collaboration is essential [19, 23, 24]. Interprofessional collaboration is defined as “collaborative practice which happens when multiple health workers from different professional backgrounds work together with patients, families, caregivers and communities to deliver the highest quality of care” [25]. It has been identified that good collaboration improves collaborative care, action continuity, relationship improvement, saves time, and promotes lifelong learning [26]. To achieve interprofessional collaboration care must be organized and coordinated across different settings and among various providers to address the present risk factors. However, due to the lack of interprofessional collaboration, applying multifactorial FPIs in current practice appears challenging [27].

The influencing factors of interprofessional collaboration in primary health care have been analyzed in several literature reviews [26, 28–30]. These include improved team communication, professional roles and duties clarity, a shared vision, effective teamwork, and action plan coordination [30, 31]. Despite the critical necessity for interprofessional collaboration in multifactorial FPIs, influencing factors have not yet been assessed within this area [32]. To develop strategies that enhance interprofessional collaboration in FPIs, it is essential to comprehend the factors that influence interprofessional collaboration. Therefore, this study aimed to provide an overview of influencing factors of interprofessional collaboration in multifactorial FPIs for community-dwelling older adults living.

## Methods

### Design

A qualitative systematic literature research was conducted using a meta-aggregation approach to identify influencing factors of interprofessional collaboration in FPIs for community-dwelling older adults. The meta-aggregative approach aims to provide generalizable statements as recommendations to advise practitioners and policymakers [33]. The review was undertaken according to the Preferred Reporting Items for Systematic Reviews and Meta-Analyses (PRISMA) statement [34]. This review is part of the Dutch implementation research project FRIEND (Fall pRevention ImplEmentatioN stuDy).The goal of the FRIEND project is to identify successful strategies for the effective, local, and integral implementation of fall prevention in the community.

### Search method

A systematic literature search in the electronic databases Pubmed, CINAHL, and Embase was conducted in March 2022. The databases were searched for articles that included terms related to the main concepts, combined with Boolean operators: ‘fall prevention’, ‘interprofessional collaboration’, ‘facilitators and barriers’ and ‘community-dwelling older adults’. The search syntax was adapted to each database (Appendix A).

### Inclusion criteria

Articles were eligible for this review if they described influencing factors of interprofessional collaboration in multifactorial FPIs for community-dwelling older adults. Articles were included if: 1) the FPI consisted of a multifactorial approach, including a physical exercise program (mobility, muscle strength and/or balance); 2) at least two different professions/disciplines in the community were involved in the FPI; 3) the FPIs were provided to community-dwelling older adults aged 65 years and older; 4) influencing factors that affect interprofessional collaboration were described; 5) the design of the articles was qualitative (e.g. qualitative study, review, meta-analysis, meta-ethnography, case study); 6) the article was written in Dutch or English and 7) the article was available in full text. Articles were excluded if they described interprofessional collaboration within the setting of a hospital or nursing home.

### Study selection

All search results were uploaded to Mendeley Reference Manager for deduplication. After deduplication, two reviewers (JSCM, and one independent researcher) independently screened the titles and abstracts on eligibility after uploading the unique articles to a web application, Rayyan [35]. This was followed by an independent full-text examination of potentially eligible articles. When conflicts occurred, a third researcher (RZ) was approached to reach a consensus.

### Data extraction

The data of all included articles were extracted by one researcher (JSCM) into a standardized extraction sheet by manually documenting the requested components. This included: authors, publication year, country, the study aims, the profession of the study participants, setting, study design, data collection method, data analysis method, and identified influencing factors of interprofessional collaboration. By reviewing random extraction sheet components, one unaffiliated researcher verified the extraction.

### Methodological quality

A quality appraisal was conducted to assess the methodological quality of the included articles. Two researchers (JSCM, and one independent researcher) independently assessed the included articles using the Checklist for Qualitative Research by the Joann Briggs Institute (JBI) [36]. This checklist consists of ten questions appraising several quality aspects of a qualitative study (Table 2), such as: “Is there congruity between the stated philosophical perspective and the research methodology?” and “Are participants, and their voices, adequately represented?”. Disagreements between researchers were discussed until a consensus was reached.

### Synthesis

The synthesis aimed to summarize all identified influencing factors of interprofessional collaboration in multifactorial FPIs in the community. The influencing factors were inductively synthesized in three steps in accordance with the JBI Manual for Evidence Synthesis using a meta-aggregative approach [37].

First, all influencing factors were extracted from the studies and labelled as *findings*. Second, for the sufficiently similar findings, *categories* were developed. Third, *synthesized findings* based on two or more compiled *categories* were developed. One researcher (JSCM) performed the analysis and discussed it with a supervising researcher (RZ). Interview quotes were obtained from the included articles and were reported according to related categories and synthesized findings.

Then, following the ConQual method, all relevant findings from the articles were rated (JSCM) to express the degree to which the researchers’ interpretation was credible [38]. The level of credibility of each finding is rated using the following ranking scale:

• Unequivocal (findings accompanied by an illustration beyond reasonable doubt and therefore not open to challenge).
• Equivocal (findings accompanied by an illustration lacking clear association with it and therefore open to challenge).
• Unsupported (the data do not support findings).

### Confidence in the synthesized findings

In order to rate the confidence of the synthesized findings in the current review study, the ConQual methodology was used [37]. Within this methodology, the dependability of the included studies’ individual findings and the credibility of the synthesized findings in the current review study were ranked and combined. This resulted in a rating of confidence in the synthesized findings, into confidence levels of High, Moderate, Low, and Very low, according to the principles of the Grading of Recommendations Assessment, Development and Evaluation (GRADE) working group. The following five questions from the JBI Checklist for Qualitative Research were used to rank the dependability of the findings in each individual study:

- 1. Is there congruity between the research methodology and the research question or objectives?
- 2. Is there congruity between the research methodology and the methods used to collect data?
- 3. Is there congruity between the research methodology and the representation and analysis of data?
- 4. Is there a statement locating the researcher culturally or theoretically?
- 5. Is the researcher’s influence on the research, and vice-versa, addressed?

All individual findings across the included studies started at the highest level. If four to five of the answers to these questions were ‘Yes’ for an individual finding, the level of dependability of that finding remained at the highest level. If two to three responses were ‘Yes’, the dependability level of the individual finding was downgraded by one level. In all other cases, the individual finding was downgraded by two levels, resultingin a level of downgrading the dependability.

Next, the credibility of the synthesized findings in the current study was ranked. Downgrading the credibility was based on the aggregate level of dependability from across the individual findings. Downgrading for credibility occurred when not all findings within a synthesized finding were considered unequivocal. The synthesised finding was downgraded one level for a mix of unequivocal/equivocal findings.For equivocal findings, the synthesised finding was downgraded two levels. For equivocal/unsupported findings, it was downgraded three levels, and for unsupported findings only, it was downgraded four levels. This resulted in a level of downgrading the credibility.

A combination of the level of downgrading the dependability and credibility led to a ConQual score (High, Moderate, Low, Very Low).

## Results

### Study selection

**Fig. 1:**
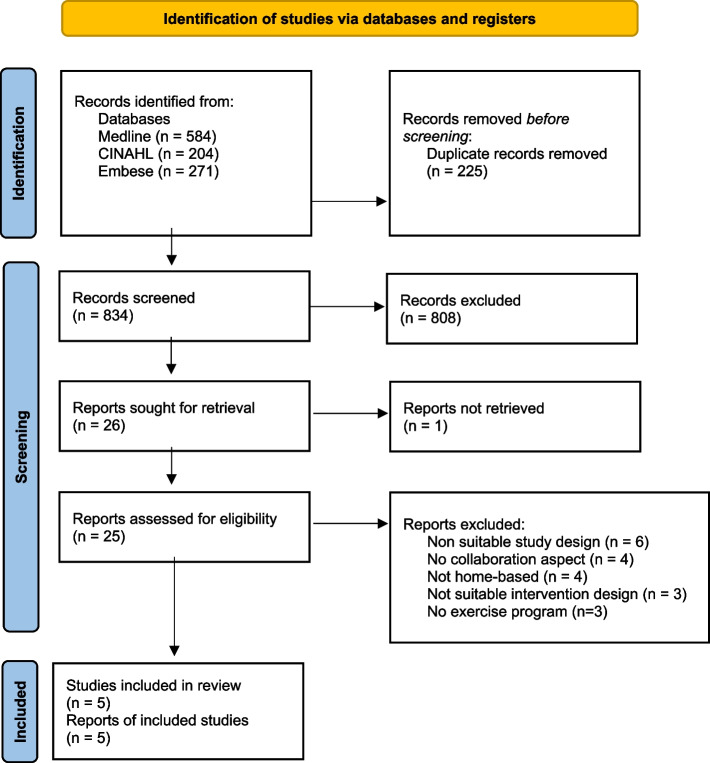
Flowchart of included articles

A total of 1.059 articles were found in a comprehensive literature search. After removing duplicates, 834 articles were assessed for relevance by title and abstract. Reasons for exclusion were, among others, that the article focused exclusively on risk assessment/screening instead of FPI, or the setting did not comply with the in- and exclusion criteria (e.g. hospital setting). This resulted in 26 potentially eligible articles. These eligible articles were sought for retrieval, of which one report was not retrieved [39]. After full-text screening, 20 articles were excluded. Six articles did not meet the inclusion criteria based on the study design [24, 40–44]. Four articles did not contain the collaboration aspect [45–48]. Four articles were not focused on community-dwelling older adults [49–52]. Three articles did not contain an exercise program [53–55]. Three articles did not include a multifactorial FPI [56–58]. This resulted in a total of five included articles [23, 59–62]. Figure 1 depicts the flow chart for the article selection process.

### Study characteristics and influencing factors of interprofessional collaboration

Table 1 lists the included articles' characteristics and identified influencing factors of interprofessional collaboration. A qualitative design was used in four of the five articles [23, 59, 61, 62]. The other article described a mixed-method design containing a qualitative aspect [60]. Semi-structured or in-depth interviews were performed in four of the articles [59–62], while the other article conducted focus groups as part of the data collection [23]. Professionals from multiple health-related disciplines were included in all included articles [23, 59–62]. Physical therapists were included in all articles [23, 59–62], and the other participating professions were nurses, occupational therapists, dieticians, case managers, general practitioners, podiatrists, exercise physiologists and rehabilitation assistants. The sample size in the articles ranged from eight to fifteen participants.

**Table 1:** Study characteristics of the included articles

Author (year); country	Aim	Participants (n)	Setting	Design and data collection	Method of analysis	Influencing factors regarding interprofessional collaboration
**Baxter et al. (2009); Canada** [23]	To describe the experiences of five different health care professionals as they participated in an interprofessional team approach to care for the frail older adult living at home and at risk of falling	Registered Nurse (2) Physiotherapist (2) Occupational Therapist (2) Nutritionist/Dietician (1) Case Manager (2)	Community Care Access Center	Exploratory descriptive qualitative design using focus groups	Thematic analysis	Understanding roles and responsibilities Feeling free to address issues Developing personal relationships Communication Working towards a common goal Gathering and sharing information Organizational support
**Middlebrook et al. (2012); Australia** [59]	To investigate the processes involved for private occupational therapists (OTs) and physiotherapists (PTs) to implement Medicare items from the Enhanced Primary Care (EPC) program within their practice, for the purpose of falls prevention interventions for older people	Occupational Therapist (4) Physical Therapist (4)	Service providers within the EPC program across the Sydney area and the Hunter region	Qualitative design with a grounded theory approach using semi-structured interviews	Grounded theory	Communication The importance of a collaborative approach Excessive paperwork and interprofessional reports Inadequacy of fee’s
**Amacher et al. (2016); Switzerland** [60]	To explore the perceived benefits and barriers of an evidence-based, home-based pilot FPP among the involved seniors, general practitioners (GPs), home care nurses (HCNs) and physiotherapists (PTs), in order to develop tailored implementation strategies	General practitioners (4) Home care nurses (4) Physiotherapists (4)	Service providers in urban and rural regions providing care to community-dwelling older adults	Mixed-method design using semi-structured interviews and questionnaires	Deductive content analysis	Doubts about the role of health care providers Overlapping skills Unclear reports and unsatisfactory information flows Invest in interprofessional aim
**Liddle et al. (2018); Australia** [61]	To explore how AHPs were making fall prevention practice routine in primary care and the factors that influenced their fall prevention practice	Physiotherapist (6) Occupational therapist (4) Exercise physiologist (2) Podiatrist (3)	Primary care settings	Explorative qualitative approach using in-depth interviews	Thematic analysis	Role clarity Overlapping skills and experiences Value of an interprofessional approach Communication should not be limited Receiving information Funding system
**Killingback et al. (2021); United Kingdom** [62]	To explore the views of healthcare practitioners involved in falls prevention in understanding how they support older people in self-managing falls and the potential for a transition pathway from NHS-exercise based falls interventions to community-run exercise programs	Physiotherapist (3) Rehabilitation assistant (3) Nurse (2)	An organization which is commissioned to provide falls rehabilitation in the North East of England	Explorative qualitative approach using semi-structured interviews	Inductive thematic analysis	Exchanging knowledge and expertise through discussions Weekly meetings to discuss patients Appreciation of the diversity of disciplines

### Methodological quality

A detailed assessment and understanding of each study’s quality was provided (Table 2). Four studies [23, 59–61] were scored positively on all items, except for item 6 and item 7. Only one study was scored positively on these two items; Killingback et al. (2021) authors stated that the researchers were experienced physical therapists who held views and assumptions about falls rehabilitation which may have influenced the research process [62]. The study by Middlebrook et al. (2012) was the only study to be scored negatively on the item on the adequate representation of voices of the participants due to a lack of provided illustrations from the data [59].

**Table 2:**
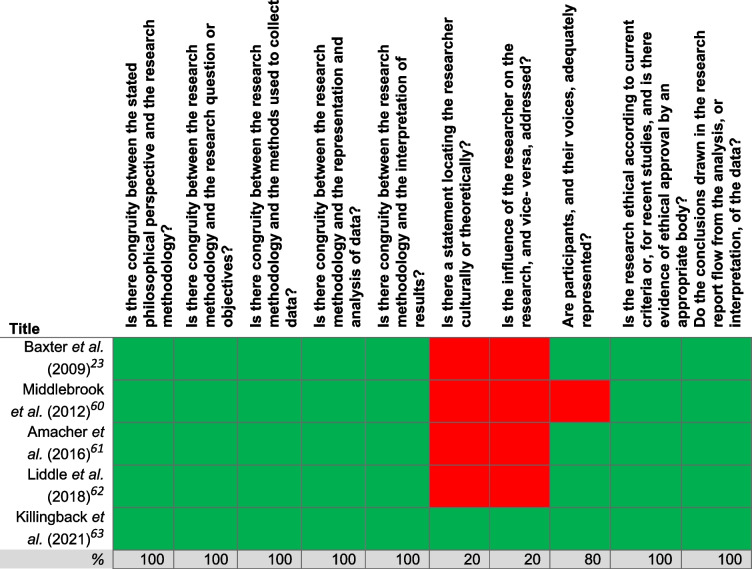
Methodological appraisal according to the checklist for qualitative research by the Joann Briggs Institute

### Synthesized findings

Analysis of the included studies resulted in 31 findings covering the identified influencing factors for interprofessional collaboration, which were summarized in ten categories (Table 3). The categories were further synthesized into five synthesized findings (Table 4). The rating of the credibility of the findings is described in Table 3. The five synthesized findings were: communication, role clarity, information sharing, organization and interprofessional aim.

**Table 3:** Analysis of the findings from the included articles, and emerging categories

**Author (year)**	**Findings**	**Categories**	1. Barriers to communication	2. Facilitators to communication	3. Understanding roles	4. Overlapping skills	5. Barrier to information sharing	6. Facilitators to information sharing	7. Work environment	8. Reimbursement	9. Working with a clear aim	10. Value of teamwork
**Baxter et al*.* (2009) **[23]	Understanding Roles (UE)				x							
	Understanding own and team members’ responsibilities (US)				x							
	Feeling free to address issues (US)								x			
	Developing personal relationships (US)								x			
	Way of communication (face to face, e-mail, telephone calls) (UE)			x								
	Communication was enhanced through teamwork (E)			x								
	Finding time to communicate (UE)		x									
	Working together towards a goal (US)										x	
	Gathering information together (UE)							x				
	Sharing information together (UE)							x				
	Organizational support (UE)								x			
**Middlebrook et al*.* (2012) **[60]	Limited verbal communication (UE)		x									
	Understanding importance of collaborative approach (E)											x
	Understanding roles (US)				x							
	Paperwork was excessive (UE)						x					
	Interprofessional reports (E)						x					
	Inadequacy of fee’s (UE)									x		
**Amacher et al*.* (2016) **[61]	Doubts about role in multidisciplinary approach (E)				x							
	Overlapping skills (UE)					x						
	Receiving unclear reports (UE)						x					
	Lack of clarity regarding the aim of FPI (US)										x	
	Unsatisfactory information flow (US)						x					
**Liddle et al*.* (2018) **[62]	Role clarity (UE)				x							
	Overlap in skills and experience across disciplines (UE)					x						
	Value of other professionals’ addition (UE)											x
	Communication was limited (E)		x									
	Receiving information about clients (US)							x				
	Reimbursed for fall prevention (UE)									x		
**Killingback et al*.* (2021) **[63]	Exchanging knowledge and expertise through discussions (UE)			x								
	Weekly meetings to discuss patients (UE)						x					
	Appreciation diversity of disciplines (US)											x

*UE* Unequivocal, *E* Equivocal, *US* Unsupported

Unequivocal (findings accompanied by an illustration that is beyond reasonable doubt and; therefore not open to challenge)

Equivocal (findings accompanied by an illustration lacking clear association with it and therefore open to challenge)

Unsupported (findings are not supported by the data)

**Table 4:** Analysis of the categories into synthesized findings, with supporting quotes from the included articles

Categories	Synthesized findings	Quote’s
**1. Barriers to communication**	Communication	*“If I’ve been seeing somebody for a couple of weeks… I’m struggling with how to get the patient to progress…then I’ll bring that patient to the meeting and say, ‘Does anybody suggest anything? Am I missing something?’So, it’s quite nice to talk it through with people, there might be something really simple and obvious, there’s a wealth of knowledge and expertise.”* [62] *“It was really difficult for us to keep up with talking about each and every one of them [clients] every month.”* [23]
**2. Facilitators to communication**		
**3. Understanding roles**	Role clarity	*“Consultingin general is very important to us HCNs. We always per-form a medical diagnostic screening and look also forthese things. (…) … and consulting (regarding facility).(We say):“You have this carpet”, then we solve this(problem)or search for solutions. Also the risk of fallingin the shower.”* [60]
**4. Overlapping skills**		*What I am enjoying about this group is that I am learning about all the different disciplines. I didn’t know a lot about the public health nursing so it was really good to learn about the different roles, and more specifically to clients in what they do so it is really positive for the community. And then physio, OT, and nutrition when they collaborate together. I learn more about what they actually do, especially with their different testings.”* [23]
**5. Barriers to information sharing**	Information sharing	*“With this particular gentleman, he does not want all these questions. I mean for all four of us to be asking him questions, so we talked last week maybe just having one or two of us to ask or maybe just one of us having to ask those questions.”* [23] *“It was put about that GPs are informed, but however, our GPs did not really have a clue. (…) But the project flyer (previously mentioned) was helpful then.”* [60]
**6. Facilitators to information sharing**		
**7. Work environment**	Organization	*“Our professional leaders are at least always sort of checking in with us to make sure everything is going okay and we have any issues that need to be taken to the working committee. Very supportive. The pressure of seeing our regular caseload but recognize too that we’ve got to see our new clients and it takes a period of time so they know when to back off with some of the pressure.”* [23] *“I still haven’t quite got my head around how it all works, the intricacies of all these new systems they have in place.”* [61]
**8. Reimbursement**		
**9. Working with a clear aim**	Interprofessional aim	*“…it’s mostly physios who send people through because they know that these people need to be motivated in another way and just giving them exercises is not enough. They need to get them to think through the issues … so they send them to me, and then they get to … consolidate what the physios been doing.”* [61]
**10. Value of teamwork**

- Synthesized finding 1: Communication

This theme is composed of six findings and two categories (communication barriers; Facilitators to communication). It relates to the way communication influences interprofessional collaboration. When communication was limited, it was found to be a barrier to collaboration.

“It was really difficult for us to keep up with talking about each and every one of them [clients] every month.” [23].

Specific ways of interpersonal communication, such as face-to-face, through e-mails or telephone calls, were perceived facilitators. Also, when knowledge and expertise were shared through discussions, it was recognized as facilitating interprofessional collaboration.

“If I’ve been seeing somebody for a couple of weeks… I’m struggling with how to get the patient to progress…then I’ll bring that patient to the meeting (…) there’s a wealth of knowledge and expertise.” [62].

- Synthesized finding 2: Role clarity

This theme was created from seven findings and two categories (Understanding roles; Overlapping skills), identifying how role clarity influences interprofessional collaboration. Understanding each others’ role well within a team, was recognized to be a facilitator.

“What I am enjoying about this group is that I am learning about all the different disciplines (…) And then physio, OT, and nutrition when they collaborate together. I learn more about what they actually do, especially with their different testings.” [23].

The fact that areas of skill in this profession overlap may have prompted reservations about interprofessional collaboration.

“Consulting in general is very important to us HCNs. We always perform a medical diagnostic screening and look also for these things. (…) … and consulting (regarding facility). (We say): “You have this carpet”, then we solve this(problem) or search for solutions.” [60].

- Synthesized finding 3: Information sharing

This theme was created based on eight findings and two categories (Barriers to information sharing; Facilitators to information sharing). It corresponds to how professionals share information within the team and how that relates to collaboration, and it can act both as a barrier and a facilitator. Barriers were excessive paperwork or meetings, or when the shared reports were unclear.

“It was put about that GPs are informed, but however, our GPs did not really have a clue.” [60].

However, whenever the way sharing and gathering data were satisfactory, this was perceived as a facilitator to interprofessional collaboration.

*“With this particular gentleman, he does not want all these questions. I mean for all four of us to be asking him questions, so we talked last week maybe just having one or two of us to ask or maybe just one of us having to ask those questions.”* [23].

- Synthesized finding 4: Organization

Five findings and two categories (Work environment; Reimbursement) formed the basis for this theme. It relates to the way an organization can influence interprofessional collaboration. The work environment plays a part in interprofessional collaboration. This was a perceived facilitator when participants felt free to address issues, developed personal relationships, and received organizational support.

“Our professional leaders are at least always sort of checking in with us to make sure everything is going okay and we have any issues that need to be taken to the working committee. Very supportive.” [23].

Whenever the way of reimbursement was unclear, this could play a part between professionals, and hinder collaboration.

“I still haven’t quite got my head around how it all works, the intricacies of all these new systems they have in place.” [61].

- Synthesized finding 5: Interprofessional aim

This theme was composed of five findings and two categories (Working with a clear aim; Value of teamwork) and relates to having a common interprofessional aim of the professionals participating in the multifactorial FPIs and how this influences the collaboration. Participants described that working with a clear aim has an impact on the collaboration.

Also, the way professionals valued the interprofessional collaboration seemed to have an impact. When professionals appreciated the team members’ addition, this was found to have a positive impact on collaboration.

“…it’s mostly physios who send people through because they know that these people need to be motivated in another way and just giving them exercises is not enough.” [61].

### Confidence levels of synthesized findings

Table 5 shows the results of the confidence levels of the synthesized findings. The synthesized finding “Communication” had the highest confidence level, which was determined as “Low”. The confidence level of the other synthesized findings appeared to be “Extremely Low”. The main reasons for the extremely low confidence levels were the credibility of the findings.

**Table 5:** Confidence levels of synthesized findings

Synthesized finding	Dependability	Credibility	Confidence level
Communication	Downgrade -1^a^	Downgrade -1^b^	Low
Role clarity	Downgrade -1^a^	Downgrade -3^d^	Extremely low
Information sharing	Downgrade -1^a^	Downgrade -3^d^	Extremely low
Organization	Downgrade -1^a^	Downgrade -2^c^	Extremely low
Interprofessional aim	Downgrade -1^a^	Downgrade -3^d^	Extremely low

^a^Downgraded one level due to common dependability issues across the included studies (the majority of studies had no statement locating the researcher and no acknowledgement of their influence on the research)

^b^Downgraded one leve due to a mix of unequivocal and equivocal findings

^c^Downgraded two levels due to a mix of unequivocal and unsupported findings

^d^Downgraded three levels due to a mix of plausible/unsupported findings

## Discussion

This review aimed to provide an overview of the influencing factors of interprofessional collaboration in multifactorial FPIs. This literature review resulted in 31 findings, ten categories, and five synthesized findings: communication, role clarity, information sharing, organization and interprofessional aim.

To our knowledge, this review is the first to provide a comprehensive summary of findings on this topic specifically in the context of multifactorial FPIs. Knowledge in this area is considerably relevant given the multifactorial nature of falls which demands an integrated, multidomain approach including both health and social care [63]. This integrated approach to health issues in general is essential since a growing number of older adults are living with complex and comprehensive healthcare needs [25, 32, 64, 65]. In order to offer high-quality care within this integrated approach to health care, effective interprofessional collaboration is essential [66]. However, numerous health systems worldwide need to be more cohesive and able to manage unmet health needs [25]. For that reason, interprofessional collaboration has been the main research topic of many studies in the field of health and social care. Research identifying challenges of and facilitators to interprofessional collaboration within various scopes of integrated care (e.g. chronic care, primary care, social care, community care, inpatient care) have been continued to accumulate [64, 65, 67–69].

Given the relatively similar focus within integrated care compared to multifactorial falls-related care, corresponding results on interprofessional collaboration have been found between the current review and other studies. This emphasizes that some aspects of interprofessional collaboration are essential to consider within any health and social care setting. For example, results in the current review show that effective communication between professionals involved in multifactorial FPIs is an essential factor in enhancing interprofessional collaboration. Also, defining roles and responsibilities (i.e. role clarity) within an interprofessional team is important. The need for effective communication and role clarity has been supported by a broad base of literature examining influencing factors for interprofessional collaboration in different healthcare settings [32, 65, 70, 71]. A recent review indicated that a ‘lack of clear role boundaries and responsibilities’ and ‘poor communication’ were amongst the most frequently identified barriers across different types of collaborations in primary care [32]. Additionally, effective communication have been shown to be a crucial competency for well-functioning interprofessional collaboration, since it increases awareness of each other’s skills and roles [72]. Effective communication encompasses a wide range of goals, strategies and purposes, such as good formal and information communication, skilful negotiation to overcome differences in viewpoints and the ability to adjust the language to the target audience [73]. Concerning role clarity, explicitly defining the function of each member in the interprofessional collaboration and his/her contribution to the collaboration may lead to a more smooth functioning between professionals [74].

Furthermore, regarding information sharing, professionals in the included articles expressed the need to share and receiving clear client reports in interprofessional collaboration. This has been highlighted in previous research as well; failing to pass along required information to be enabled to provide optimal care reduced collaboration, since it leads to individual team members to collect information from other sources themselves [75, 76]. Transferring information between team members could be provided during regular team meetings, which enables professionals to discuss issues that arise [75, 76]. However, when the information-sharing process is excessive, e.g. too many meetings are scheduled, this could become a barrier [32]. This particular finding was also identified in the included articles in this review, where professionals mentioned that excessive paperwork and meetings were perceived barriers to interprofessional collaboration.

Additionally, in the current review, professionals described the necessity of having a clear interprofessional aim, including shared vision, common team goals, and valuation of each members’ contribution. This may enable teams to set clear directions, which may lead to teams being action-oriented. Also, the process of setting clear goals could contribute to enhancing role clarity, as team members need to indicate what their specific part is in achieving the goals [75]. Making the time to develop a clear interprofessional aim and facilitating interprofessional collaboration in general requires time, support, effective personal relationships and an open atmosphere. Therefore, reimbursements and sufficient funding are essential [32]. However, with organizations having different financial structures, policies and funding, it is often difficult to initiate collaborative efforts when other organizations have few resources to invest in the relationship [77].

There are some limitations to this review. First, we only included five articles, which may have consequences for generalizing findings to different settings. However, the scarce evidence in this area underlines the importance of conducting this review. Second, as with all reviews, we risk missing relevant research due to interchangeable terminology, such as interdisciplinary collaboration, interprofessional collaborative practices, multi-professional cooperation and interaction within health care teams [78]. However, we attempted to identify all relevant studies by using various terms related to the main concepts of ‘interprofessional teamwork’, ‘influencing factors’, ‘fall prevention’, and ‘older individuals’. Consensus on terminology usage may result in definitions that can be used in education, research, and practice and could improve communication between sectors, settings and providers [78]. Third, the confidence level of the synthesized findings according to the ConQual methodology were low or extremely low. This could affect the implications of this review. Nevertheless, the findings of this research overlap significantly with other studies that investigated the influencing factors of interprofessional collaboration in the healthcare domain [64, 65, 67–69, 75–77], suggesting that these results are still valuable.

The main strength of this review is that factors which influence interprofessional collaboration in the context of multifactorial FPIs were thoroughly examined using rigor methodology. The JBI is a well-known institute, having a widely-used checklist for critically appraising the trustworthiness, relevance and results of included articles [37]. The ConQual methodology is also widely-used approach to establish confidence in the evidence produced by qualitative systematic reviews [38]. This resulted in a comprehensive summary of the findings. Another strength was the specificity of the aim (i.e. interprofessional collaboration within the scope of multifactorial FPIs). This focus enables the transfer of findings to professionals working collaboratively within the context of falls prevention in community-dwelling older adults. Furthermore, the inductive approach to analyzing the barriers and facilitators that arose from the included studies, allowed findings to emerge from frequent, inherent, raw data into summarized, significant themes [79]. Understanding factors that may hinder or enable interprofessional collaboration in this particular context of fall prevention allows us to then design effective implementation strategies to guide further implementation efforts. The current reviews’ results suggest that in a next step of designing implementation strategies, we should focus on the results of the synthesized findings. Future research should pay attention to designing clearly defined strategies, with the goal to ensure long-term, sustainable outcomes. Next, it is necessary to evaluate these strategies for effectiveness and specify the strategies' working mechanisms. This will increase the likelihood that the strategies will eventually lead to desired implementation outcomes, aiming at improving interprofessional collaboration in fall prevention practices [80].

## Conclusion

In conclusion, multiple barriers and facilitators influence interprofessional collaboration in multifactorial FPIs in community-dwelling older adults. These can be summarized into five overarching themes: communication, role clarity, information sharing, organization and interprofessional aim. This review fills an important gap in the literature by providing a comprehensive overview of synthesized findings, which can be used to develop effective implementation strategies. Applying these implementation strategies will help to improve interprofessional collaboration between health and social care professionals working in multifactorial FPIs in the community.

## Supplementary Information

**Additional file 1.**

## Data Availability

The datasets used and/or analyzed during the current study are available from the corresponding author on reasonable request.

## Abbreviations

FPI: Fall prevention interventions
PRISMA: Preferred Reporting Items for Systematic Reviews and Meta-Analyses
JBI: Joann Briggs Institute
GRADE: Grading of Recommendations Assessment, Development and Evaluation
