# Supplementary material for: Influencing factors of interprofessional collaboration in multifactorial fall prevention interventions: a qualitative systematic review
Source: BMC Prim Care. 2023 May 16;24:116. doi: 10.1186/s12875-023-02066-w (PMC10186662; doi:10.1186/s12875-023-02066-w)
Supplement: Supplementary file 1 — Additional file 1. [file 12875_2023_2066_MOESM1_ESM.docx]

Appendix A. Search queries

| Search | Query MEDLINE |
| --- | --- |
| #1 Fall prevention | ((((((((((((((((((((((Fall* prevent*[Title/Abstract]) OR (Fall* prevent* program*[Title/Abstract])) OR (Fall* prevent* protocol*[Title/Abstract])) OR (Fall* prevent* training*[Title/Abstract])) OR (Fall* prevent* training* program*[Title/Abstract])) OR (Fall* prevent* intervent*[Title/Abstract])) OR (Fall* prevent* intervent* program*[Title/Abstract])) OR (Fall* prevent* exercise*[Title/Abstract])) OR (Fall* prevent* exercise* program*[Title/Abstract])) OR (Fall* prevent* strategie*[Title/Abstract])) OR (Fall* prevent* plan*[Title/Abstract])) OR (Fall* program*[Title/Abstract])) OR (Fall* protocol*[Title/Abstract])) OR (Fall* training*[Title/Abstract])) OR (Fall* training* program*[Title/Abstract])) OR (Fall* intervent*[Title/Abstract])) OR (Fall* intervent* program*[Title/Abstract])) OR (Fall* exercise*[Title/Abstract])) OR (Fall* exercise* program*[Title/Abstract])) OR (Fall* stategie*[Title/Abstract])) OR (Fall* plan*[Title/Abstract])) OR (Exercise-based fall* prevent*[Title/Abstract])) OR (Exercises-based fall* prevent*[Title/Abstract]) |
| #2 Interprofessional collaboration | ("Intersectoral Collaboration"[Mesh]) OR (((((((((((((((((((((((((Collab*, Intersectoral) OR (Intersectoral Collab*)) OR (Intersectoral Cooperat*)) OR (Cooperat*, Intersectoral)) OR (Collab*)) OR (Cooperat*)) OR (Interprof* collab*)) OR (Inter-prof* collab*)) OR (Interprof* cooperat*)) OR (Inter-prof* cooperat*)) OR (Multidisciplinary collab*)) OR (Multi-disciplinary collab*)) OR (Multidisciplinary cooperat*)) OR (Multi-disciplinary cooperat*)) OR (Interdisciplinary collab*)) OR (Inter-disciplinary collab*)) OR (Interdisciplinary cooperat*)) OR (Inter-disciplinary cooperat*)) OR (Intersectoral collab*)) OR (Inter-sectoral collab*)) OR (Intersectoral cooperat*)) OR (Inter-sectoral cooperat*)) OR (Prof* collab*)) OR (Prof* cooperat*)) OR (Team*)) |
| #3 Facilitators and barriers | ((((((((((((((((((Facilitat* barrier*) OR (Facilitat*)) OR (Barrier*)) OR (Enhanc* factor*)) OR (Enhanc* component*)) OR (Enhanc*)) OR (Factor* influenc*)) OR (Influencing component*)) OR (Benefi*)) OR (Block*)) OR (Obstacle*)) OR (Hinder*)) OR (Enabl*)) OR (Constrain*)) OR (Opportunit*)) OR (Chanc*)) OR (Associat* factor*)) OR (Moderat* factor*)) OR (Influenc*) |
| #4 Older adults | (((((((((("Aged, 80 and over"[Mesh]) OR ("Aged"[Mesh])) OR (Older adult*)) OR (Elderly)) OR (Aging)) OR (Senior*)) OR (Older people*)) OR (Aged)) OR (65+)) OR (Oldest old)) OR (Elderly people*) |

| Search | Query Embase |
| --- | --- |
| #1 Fall prevention | ('Fall* prevent*':ti,ab,kw OR 'Fall* prevent* program*':ti,ab,kw OR 'Fall* prevent* protocol*':ti,ab,kw OR 'Fall* prevent* training*':ti,ab,kw OR 'Fall* prevent* training* program*':ti,ab,kw OR 'Fall* prevent* intervent*':ti,ab,kw OR 'Fall* prevent* intervent* program*':ti,ab,kw OR 'Fall* prevent* exercise*':ti,ab,kw OR 'Fall* prevent* exercise* program*':ti,ab,kw OR 'Fall* prevent* strategie*':ti,ab,kw OR 'Fall* prevent* plan*':ti,ab,kw OR 'Exercise-based fall* prevent*':ti,ab,kw OR 'Exercises-based fall* prevent*':ti,ab,kw OR 'Fall* program*':ti,ab,kw OR 'Fall* protocol*':ti,ab,kw OR 'Fall* training*':ti,ab,kw OR 'Fall* training* program*':ti,ab,kw OR 'Fall* intervent*':ti,ab,kw OR 'Fall* intervent* program*':ti,ab,kw OR 'Fall* exercise*':ti,ab,kw OR 'Fall* exercise* program*':ti,ab,kw OR 'Fall* stategie*':ti,ab,kw OR 'Fall* plan*':ti,ab,kw OR 'fall prevention'/exp) |
| #2 Interprofessional collaboration | ('Collab*, Intersectoral' OR 'Intersectoral Collab*' OR 'Intersectoral Cooperat*' OR 'Cooperat*, Intersectoral' OR 'Collab*' OR 'Cooperat*' OR 'Interprof* collab*' OR 'Inter-prof* collab*' OR 'Interprof* cooperat*' OR 'Inter-prof* cooperat*' OR 'Multidisciplinary collab*' OR 'Multi-disciplinary collab*' OR 'Multidisciplinary cooperat*' OR 'Multi-disciplinary cooperat*' OR 'Interdisciplinary collab*' OR 'Inter-disciplinary collab*' OR 'Interdisciplinary cooperat*' OR 'Inter-disciplinary cooperat*' OR 'Intersectoral collab*' OR 'Inter-sectoral collab*' OR 'Intersectoral cooperat*' OR 'Inter-sectoral cooperat*' OR 'Prof* collab*' OR 'Prof* cooperat*' OR 'Team*' OR 'interprofessional collaboration'/exp OR 'intersectoral collaboration'/exp) |
| #3 Facilitators and barriers | ('Facilitat* barrier*' OR Facilitat* OR Barrier* OR 'Enhanc* factor*' OR 'Enhanc* component*' OR Enhanc* OR 'Factor* influenc*' OR 'Influencing component*' OR Benefi* OR Block* OR Obstacle* OR Hinder* OR Enabl* OR Constrain* OR Opportunit* OR Chanc* OR 'Associat* factor*' OR 'Moderat* factor*' OR 'Influenc*' OR 'barrier'/exp OR 'facilitator'/exp) |
| #4 Older adults | ('Older adult*' OR Elderly OR Aging OR Senior* OR 'Older people*' OR Aged OR '65+' OR 'Oldest old' OR 'Elderly people*' OR 'aged'/exp) |

| Search | Query CINAHL |
| --- | --- |
| #1 Fall prevention | ((TI Fall* prevent*) OR (AB Fall* prevent*)) OR ((TI Fall* prevent* program*) OR (AB Fall* prevent* program*)) OR ((TI Fall* prevent* protocol*) OR (AB Fall* prevent* protocol*)) OR ((TI Fall* prevent* training*) OR (AB Fall* prevent* training*)) OR ((TI Fall* prevent* training* program*) OR (AB Fall* prevent* training* program*)) OR ((TI Fall* prevent* intervent*) OR (AB Fall* prevent* intervent*)) OR ((TI Fall* prevent* intervent* program*) OR (AB Fall* prevent* intervent* program*)) OR ((TI Fall* prevent* exercise*) OR (AB Fall* prevent* exercise*)) OR ((TI Fall* prevent* exercise* program*) OR (AB Fall* prevent* exercise* program*)) OR ((TI Fall* prevent* strategie*) OR (AB Fall* prevent* strategie*)) OR ((TI Fall* prevent* plan*) OR (AB Fall* prevent* plan*)) OR ((TI Fall* program*) OR (AB Fall* program*)) OR ((TI Fall* protocol*) OR (AB Fall* protocol*)) OR ((TI Fall* training*) OR (AB Fall* training*)) OR ((TI Fall* training* program*) OR (AB Fall* training* program*)) OR ((TI Fall* intervent*) OR (AB Fall* intervent*)) OR ((TI Fall* intervent* program*) OR (AB Fall* intervent* program*)) OR ((TI Fall* exercise*) OR (AB Fall* exercise*)) OR ((TI Fall* exercise* program*) OR (AB Fall* exercise* program*)) OR ((TI Fall* strategie*) OR (AB Fall* strategie*)) OR ((TI Fall* plan*) OR (AB Fall* plan*)) OR ((TI Exercise-based fall* prevent*) OR (AB Exercise-based fall* prevent*)) OR ((TI Exercises-based fall* prevent*) OR (AB Exercises-based fall* prevent*)) |
| #2 Interprofessional collaboration | ((MH “Collaboration”) OR Collab*, Intersectoral OR Intersectoral Collab* OR Intersectoral Cooperat* OR Cooperat*, Intersectoral OR Collab* OR Cooperat* OR Interprof* collab* OR Inter-prof* collab* OR Interprof* cooperat* OR Inter-prof* cooperat* OR Multidisciplinary collab* OR Multi-disciplinary collab* OR Multidisciplinary cooperat* OR Multi-disciplinary cooperat* OR Interdisciplinary collab* OR Inter-disciplinary collab* OR Interdisciplinary cooperat* OR Inter-disciplinary cooperat* OR Intersectoral collab* OR Inter-sectoral collab* OR Intersectoral cooperat* OR Inter-sectoral cooperat* OR Prof* collab* OR Prof* cooperat* OR Team*) |
| #3 Facilitators and barriers | (Facilitat* barrier* OR Facilitat* OR Barrier* OR Enhanc* factor* OR Enhanc* component* OR Enhanc* OR Factor* influenc* OR Influencing component* OR Associat* factor* OR Chanc* OR Moderat* factor* OR Opportunit* OR Influenc* OR Enabl* OR Constrain* OR Benefi* OR Block* OR Obstacle* OR Hinder*) |
| #4 Older adults | ((MH "Aged") OR (MH "Aged, 80 and Over") OR Older adult* OR Elderly OR Aging OR Senior* OR Older people* OR Aged OR 65+ OR Oldest old OR Elderly people*) |
